# Whole-Brain Atrophy Rate in Idiopathic Parkinson's Disease, Multiple System Atrophy, and Progressive Supranuclear Palsy

**DOI:** 10.1155/2016/9631041

**Published:** 2016-04-14

**Authors:** C. Guevara, K. Bulatova, G. J. Barker, G. Gonzalez, N. Crossley, M. J. Kempton

**Affiliations:** ^1^Facultad de Medicina, Universidad de Chile, Santos Dummont 999, Santiago, Chile; ^2^Department of Neuroimaging, Institute of Psychiatry, Psychology & Neuroscience, King's College London, London SE5 8AF, UK

## Abstract

In multiple system atrophy (MSA) and progressive supranuclear palsy (PSP), the absence of surrogate endpoints makes clinical trials long and expensive. We aim to determine annualized whole-brain atrophy rates (a-WBAR) in idiopathic Parkinson's disease (IPD), MSA, and PSP. Ten healthy controls, 20 IPD, 12 PSP, and 8 MSA patients were studied using a volumetric MRI technique (SIENA). In controls, the a-WBAR was 0.37% ± 0.28 (CI 95% 0.17–0.57), while in IPD a-WBAR was 0.54% ± 0.38 (CI 95% 0.32–0.68). The IPD patients did not differ from the controls. In PSP, the a-WBAR was 1.26% ± 0.51 (CI 95%: 0.95–1.58). In MSA, a-WBAR was 1.65% ± 1.12 (CI 95%: 0.71–2.59). MSA did not differ from PSP. The a-WBAR in PSP and MSA were significantly higher than in the IPD group (*p* = 0.004 and *p* < 0.001, resp.). In PSP, the use of a-WBAR required one-half of the patients needed for clinical scales to detect a 50% reduction in their progression. In MSA, one-quarter of the patients would be needed to detect the same effect. a-WBAR is a reasonable candidate to consider as a surrogate endpoint in short clinical trials using smaller sample sizes. The confidence intervals for a-WBAR may add a potential retrospective application for a-WBAR to improve the diagnostic accuracy of MSA and PSP versus IPD.

## 1. Introduction

There is a need to characterize disease progression in idiopathic Parkinson's disease (IPD), multiple system atrophy (MSA), and progressive supranuclear palsy (PSP) to test the effectiveness of disease-modifying interventions. PSP and MSA can be misdiagnosed with IPD or vice versa at the initial stages of the disease and differential diagnosis can be challenging, although a number of neuroimaging techniques allow a partial distinction among the diseases [1]. Clinical trials for these disorders are hampered by the lack of surrogate endpoints and the unknown* tempo* of neuronal destruction. While clinical scales have been largely used to measure disease progression in therapeutic trials, they have a number of inherent limitations that reduce their sensitivity for tracking disease progression. They may show nonlinearity, floor and ceiling effects, or an inability to differentiate symptomatic changes from disease modification changes. In addition there may be influences from other disorders, behavioral fluctuations, and effects of medications [2]. These factors may increase the variability of clinical data and limit both their effectiveness as outcome measures and their utility as inputs to power calculations when generating sample size estimates for clinical trials.

Whole-brain atrophy rates (WBAR) from MRI data may be an informative way to quantify disease progression in an unbiased fashion, although to date, only three studies have used such an approach in PSP and MSA [3–5]. This approach reduces interindividual variability in brain size and morphology when baseline scans are used as reference points so that the subject acts as his or her own control.

In this study on Latin American Chilean patients, we used the Structural Image Evaluation Using Normalization of Atrophy (SIENA) [6, 7] to estimate a-WBAR (annualized whole-brain atrophy rates) in IPD, PSP, and MSA. Furthermore, we determined the associations of a-WBAR with clinical scales, explored the retrospective application of a-WBAR to differentiate MSA and PSP from IPD, and reported sample sizes per arm derived from these clinical and MRI measures for a hypothetical placebo-controlled disease-modifying trial to estimate their relative utility as primary outcomes.

## 2. Materials and Methods

### 2.1. Subjects and Clinical Assessment

Fifty participants (10 healthy controls, 20 IPD, 12 PSP, and 8 MSA patients) were recruited from the Movement Disorders Clinic at the Hospital San Juan de Dios, Santiago, Chile. Internationally established operational criteria were used to assess the diagnoses of MSA, PSP, and IPD [8–10]. Fourteen IPD patients had the tremor dominant phenotype and six had the postural instability gait disorder phenotype. Of the 12 PSP patients, eleven had the typical features of classic PSP (Richardson's syndrome) and one had an atypical profile with tremor and moderate L-DOPA responsiveness (PSP-Parkinsonism variant). Six probable MSA patients were categorised as MSA-P (predominant parkinsonian features) and two as MSA-C (predominant cerebellar features). All participants were assessed on their usual dopaminergic medication and the IPD patients were evaluated in the “on state.” The patients' demographics and clinical variables are presented in Table 1.

The clinical parameters and correlations of a-WBAR with clinical scales were explored using the 18 items of the Movement Disorder Society-Sponsored Revision of the Unified Parkinson's Disease Rating Scale (MDS-UPDRS) motor symptoms (UPDRS III) [11, 12], Hoehn and Yahr Scale (H&Y) [13], and the Clinical Global Impression for Disease Severity (CGI-S) [14]. The Mini-Mental State Examination (MMSE) was used as a measure of general cognitive function [15]. Executive function was assessed using the Frontal Assessment Battery (FAB) [16]. The mean time interval between clinical assessments was 1.03 ± 0.08 years.

### 2.2. MRI Acquisition

Between 2012 and 2014, patients underwent a MRI brain scan. MRI images were acquired on a 3.0 T Philips Medical System. Axial T_1_-weighted images of the whole-brain were obtained using a 3D inversion recovery prepared spoiled gradient echo (IR-SPGR) sequence. The following parameters were used: repetition time of 8.1 ms; echo time of 3.7 ms; inversion time of 450 ms; voxel size of 0.699 × 0.699 × 1 mm; excitation flip angle of 8°; matrix size of 248 × 226; field of view of 24 cm; and 198 axial slice of 1 mm. An experienced neuroradiologist (GG) assessed the MRI scans of every patient to rule out gross anatomical abnormalities. Patients underwent a second MRI brain scan at the time of the last study visit (12 months after the baseline scan). Subjects were included in the study if they had two MRI scans of adequate quality and the brain extraction step in SIENA functioned correctly. None of the MRI images included in this study showed any structural abnormalities other than atrophy-related changes. These inclusion criteria were assessed by a visual inspection of the raw and processed data for each patient scan. For both the baseline and the follow-up assessments, the clinical data and MRI scans were acquired within one week of each other. The mean scan interval was 1.04 ± 0.07 years.

### 2.3. Data Analysis

All of the images were converted in NIFTI format in preparation of processing using SIENA. Before further processing, all of the data were anonymized by removing any references to the patients' names from the image headers and ensuring that the file names were based on a unique ID rather than any of the patients' personal details, including their clinical group. The SIENA processing algorithm has been validated and described in detail elsewhere [7]. Briefly, the processing stages are as follows: (1) brain extraction (BET): segmentation of the brain from nonbrain tissue for each scan, followed by skull extraction; (2) registration: the segmented brain from the second (follow-up) scan is registered to that of the first (baseline) using a linear transformation; the two skull images are used as normalizing factors to constrain the scale and skew; (3) tissue type segmentation: white matter and grey matter tissues are treated as one tissue and the cerebrospinal fluid as another; (4) change analysis: in this step, the method detects the brain edge on both registered brain images and then estimates the motion of the brain surface edges. The direction of movement from the first image to second image indicates whether atrophy or growth has occurred. Finally, the percentage of global brain volume change is obtained for each subject from the mean of all of the edge point motions. SIENA has been shown to have 0.5% brain volume accuracy in longitudinal studies [6, 7].

### 2.4. Statistical Analyses

Statistical analyses of the clinical data and clinical-imaging correlations were performed using the Statistical Package for Social Sciences (SPSS, Inc., Chicago, IL, USA, version 22). The results are presented as the mean ± SD. In all cases, a two-sided *p* value of <0.05 was considered significant. Visual inspection of the data using histograms and QQ-plots was performed to check for violations of the assumption of a normal distribution. Levene's test of equal variances was used to verify the assumption of the homogeneity of variances. As a result of these checks, parametric and nonparametric statistical tests were used. One-way analysis of variance was performed for normally distributed data (age at examination, disease duration, and a-WBAR). The Tukey test was used to control for multiple testing. Because disease severity and neuropsychological measures were nonnormally distributed, they were compared between groups using a Kruskal-Wallis test, and when necessary, a* post hoc* procedure with Bonferroni correction for multiples testing (*p* values were multiplied by 4) was used to compare the four disease groups. A *χ* ^2^ test for homogeneity was used to compare the distribution of males and females across groups. The a-WBAR was calculated by dividing the WBAR values by the interscan interval in years. Clinical scores were also annualized by dividing the unit change between assessments by years. Changes in the clinical scores were assessed using Wilcoxon's signed rank test. The associations between a-WBAR and annualized changes in clinical scores were assessed using bivariate correlations and simple linear regressions.

In each group, the sample size requirements to detect a treatment difference per arm were estimated for a hypothetical placebo-controlled disease-modifying trial. Annualized changes in the clinical scores and a-WBAR were compared. All of the calculations were based on the assumption of a treatment effect corresponding to a small (20%) or a moderate (50%) reduction in the annualized change in each outcome measure, 90% power, and two-sided 0.05 level of significance according to the standard formula:

(1) $$\begin{array}{c} \text{sample size per trial arm: }{\left(\;u+v\;\right)}^{2}\times \frac{\left(2\sigma^{2}\right)}{{\left(\mu_{1}-\mu_{2}\right)}^{2}}, \end{array}$$

where *u* = 1.28 to provide 90% power, *v* = 1.96 to test at the 5% significance level, and *μ*_1_ and *μ*_2_ are the a-WBAR or mean change in clinical scores in the placebo and treatment groups. *σ* ^2^ is the common variance of the atrophy rate or of the clinical score changes in both arms. The a-WBAR and changes in the clinical scores were taken as the percentage of the difference between the control and active groups.

### 2.5. Standard Protocol Approval, Registrations, and Patient Consent

Prior to inclusion, patients gave their informed written consent to participate in the study. The study was conducted according to International Standards of Good Clinical Practice (ICH guidelines and the Declaration of Helsinki). The project was approved by the local Research Ethics Committees of San Juan de Dios Hospital, Santiago, Chile.

## 3. Results

### 3.1. Demographics, Clinical Variables, and a-WBAR (Table 1)

**Figure 1:**
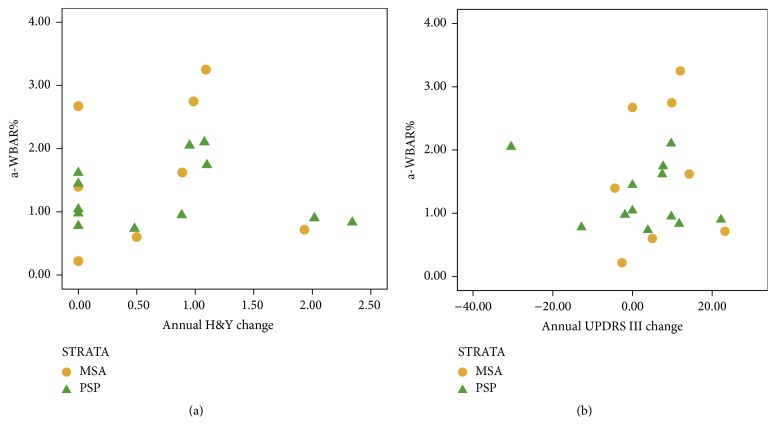
Relationships between annual changes in H&Y scale (a) and UPDRS (b) versus a-WBAR in MSA and PSP groups: 8 patients with brain atrophy showed either improvement (<0) or unchanged values (=0) on clinical measures.

There were no significant differences in age, gender, and disease duration between the groups. The PSP patients had a shorter disease duration (2.2 ± 1.5 years). The PSP and MSA patients showed greater impairment in the UPDRS III, H&Y, and CGI-S than the IPD patients. The PSP patients showed greater impairment on the cognitive measures than the IPD patients. The IPD group did not show significant overall clinical deterioration over the one-year follow-up period, as did the MSA and PSP patients on the H&Y scale and the MSA patients on the CGI-S scale (Table 2). Visual inspection of the data indicated that some of the patients showed either improvement or unchanged values on clinical measures (Figure 1). In contrast, all cases showed a brain volume loss between scans. In the controls, a-WBAR was 0.37% ± 0.28 (CI 95% 0.17–0.57), and in IPD a-WBAR was 0.54% ± 0.38 (CI 95% 0.32–0.68). The IPD patients did not differ from the controls.

**Figure 2:**
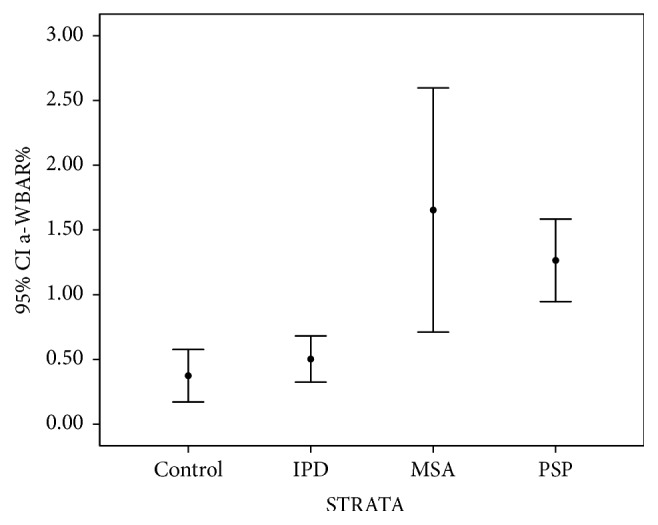
Error bars showing 95% confident intervals of a-WBAR means for each group.

In PSP, a-WBAR was 1.26% ± 0.51 (CI 95%: 0.95–1.58). In MSA, the a-WBAR was 1.65% ± 1.12 (CI 95%: 0.71–2.59). MSA did not differ from PSP. a-WBAR in the PSP and MSA patients was significantly higher compared to the IPD group (*p* = 0.004 and *p* < 0.001, resp.) (Table 1, Figure 2).

### 3.2. Relationships between the Clinical Scores and a-WBAR

No significant correlations were found between a-WBAR and annualized clinical assessments in the three disease groups.

### 3.3. Sample Size Estimates

Table 2 (columns 5 and 6) summarizes the estimates of the sample size needed to detect small (20%) or moderate (50%) reductions in the rates of a-WBAR in response to treatment and similar annualized changes in clinical scores. Using a-WBAR in PSP, 83 patients would be required to detect a small treatment effect, and 14 patients would be required to detect a moderate effect. In MSA, 234 and 38 patients would be needed to detect small and moderate treatment effects, respectively. Using clinical measurements, much larger patient cohorts would be required to detect equivalent effects.

## 4. Discussion

The research of an early biomarker of disease progression of atypical parkinsonisms is the main aim of this study. For normal ageing, the a-WBAR has been estimated to be 0.3–0.5% [4, 5, 17], as reported here (0.37%). In this longitudinal study, we found an a-WBAR of 1.26% for PSP and 1.65% for MSA, similar to previous reports [3–5]. In six autopsy-confirmed PSP cases [4], the a-WBAR (measured using the boundary shift integral (BSI) [18], a (semi)automated technique similar to SIENA) was 1.3%. In another five proven PSP cases, this figure was 1% [4, 5]; in another study, also using BSI, a-WBAR estimates were approximately 1% for both PSP and MSA based on 17 PSP cases and 9 cases with MSA-P [3]. Consistent with previous reports, no significant difference was observed in the current study between a-WBAR in PSP and MSA [3].

It is plausible that cortical structures are the main contributors to whole-brain atrophy in PSP and MSA. In PSP, neuronal loss is recognized in frontal, temporal, and limbic cortices and much less in parietal and occipital cortices [19]. Such a neuronal loss is not considered to be typical in MSA. However, Papp and Lantos described high densities of glial cytoplasmic inclusions in the supplementary and primary motor cortical areas and subjacent white matter and moderate densities of glial cytoplasmic inclusions in the premotor area, cingulate motor area, and corpus callosum in MSA [20]. In a review of 203 proven MSA cases, some degree of cortical atrophy was observed in 21% of cases [10], and* post mortem* examinations showed severe frontal atrophy [21, 22].* In vivo* data in MSA showed hypometabolism in motor, premotor, and prefrontal cortices and parietal lobes [23]. A proton magnetic resonance spectroscopy study showed a significant reduction of N-acetylaspartate/creatine in the frontal cortex [24]. Voxel-based morphometry studies have suggested that atrophy in the motor and prefrontal cortices is a common finding in MSA [25].

In levodopa-responsive IPD patients, the evidence supports the idea that motor deficits predominantly relate to the localized loss of selective dopaminergic neurons in the substantia nigra, with cortical and subcortical grey and white matter structures more preserved in comparison with those of other Parkinsonian variants. In this study, the IPD patients did not show abnormal a-WBAR; a-WBAR in IPD was higher, although still within normal limits, than previously reported (0.28%). From a clinical perspective, PSP and MSA can be misdiagnosed with IPD or vice versa at the initial stages of the disease and differential diagnosis can be challenging [26]. The confidence intervals for a-WBAR reported here may add a potential retrospective application for a-WBAR to improve the diagnostic accuracy for MSA and PSP versus IPD, particularly in their initial stages, when the clinical “plus syndrome” has not yet manifested and the response to levodopa treatment is being assessed. Thus, a-WBAR within a normal range is unlikely to be observed in PSP or MSA, but is likely to be observed in IPD patients. However, before the technique can be used to help to differentiate Parkinson plus syndromes from IPD, a greater number of patients is needed to establish a discriminatory cut-off point for these measures and to estimate sensitivity, specificity, and positive predictive values.

In MSA and PSP, the absence of surrogate endpoints makes clinical trials even more difficult, long, and expensive. For a biological marker to become a suitable surrogate endpoint for clinical trials, it should not only have low variability but also directly reflect any clinical benefit that would be observed in the underlying measure it replaces [27]. The variance in clinical scales as outcome measures is a relevant problem when the need for fast and practical clinical trials is pressing. Indeed, in both PSP and MSA (diseases that are inevitably progressive), it is possible for clinical scores to remain unchanged during the longitudinal assessment, perhaps due to measurement error or the influence of incidental factors on the patients' status at the time of clinical assessment. However, clinical assessment was performed in “on state.” This point is important for monitoring the clinical course of these diseases. Indeed, a number of patients showed unchanged or improved clinical scores at the follow-up, which is quite unusual for aggressive diseases as PSP or MSA. The clinical scores might thus be influenced by medications and did not reflect their real extent.

The relatively short clinical assessment interval in our study may explain the absence of significant associations between a-WBAR and changes in clinical scores [28, 29]. The variance in these clinical scales means that large patient samples are required to detect small changes in outcomes. Conversely, every patient (in both groups) showed whole-brain atrophy during the scan interval consistent with a progressive neurodegenerative disorder. This indicates both greater reliability and sensitivity for detecting markers of degeneration using this MRI-derived measure compared to clinical assessments.

The power analysis further addressed this point. In PSP, the use of a-WBAR would require one-half of the patients needed for UPDRS III to detect 50% of reduction in their progression. In MSA, one-quarter of the patients would be needed to detect the same effect. Because the objective of the current power analysis was to compare the theoretical performance of clinical and MRI-derived measures to track disease progression in clinical trials, we did not correct for normal ageing [30] and no allowance was made for patient dropout or unusable scan pairs. These would have certainly increased the sample size estimates, so the present measurements should be interpreted in light of these caveats. This approach does not, however, impact the WBAR estimates and the variances that we observed in both groups. Thus, the rate of brain volume loss in a one-year period is a reasonable candidate to consider as a potential surrogate endpoint.

SIENA offers several advantages compared with other quantitative MRI techniques, including a high reproducibility of results and the capability to provide a robust measurement of the global changes associated with disease conditions. SIENA has also been shown to be robust to changes in acquisition parameters, including the pulse sequence and slice thickness [6]; this is an important advantage of clinical trials, which are usually multicenter in nature.

The strength of this study consists in providing an early biomarker of disease progression of atypical parkinsonisms, which could be really helpful in future prospective studies or clinical trials. These results are based on prospective collection of clinical data with patients characterized in a uniform manner and undergoing serial MRI imaging on the same scanner. We acknowledge the relatively small number of patients with MSA and the lack of pathological confirmation. Another limitation of this this study is that we did not include the Progressive Supranuclear Palsy (PSP) Rating Scale [31] and the Unified Multiple System Atrophy Rating Scale (UMSARS) [32], which are the standard clinical research scales for those diseases. The measure used that comes closest to a comprehensive scale for these diseases is the UPDRS motor scale (UPDRS III). Factor analysis has revealed that some items of UPDRS III are useful in assessing PSP patients: axial and limb bradykinesia, tremor and rigidity, and face and speech [12]. However, oculomotor, bulbar, autonomic, and cerebellar domains are not included in UPDRS III. In addition, the H&Y scale considers mild to moderate bilateral disease and postural instability, which are early findings in MSA and PSP. Therefore, when applied to MSA and PSP, the latency to stage 3 is minimal and a “floor effect” is evident; one study on pathological proven cases found that 92% of PSP and 67% of MSA patients reached stage 3 within a year of motor onset [33]. Because of this, a Parkinson plus syndrome may be suspected if a patient reaches stage 3 rapidly after the clinical onset [34]. Consequently, the scales we report here do not fully assess the wide range of deficits in MSA and PSP, and the clinical correlations we reported can only provide information on parkinsonian symptoms and cognitive functions, not to the entire clinical status of patients. According to this, the lack of correlation between a-WBAR and clinical scores may be explained. Secondly, this methodological bias may have affected the power analysis which requires a very high number of patients for diseases having a prevalence of 4-5 cases/100.000.

In the future, it would therefore be beneficial to select subscores of clinical scales that better highlight meaningful associations between atrophy rates with the symptoms and signs affecting MSA and PSP patients.

## 5. Conclusion

a-WBAR is more sensitive to disease progression than standard clinical assessments and can be reasonably considered to be a potential surrogate endpoint in short and efficient clinical trials using significantly smaller sample sizes. Finally, a retrospective application for a-WBAR may help to differentiate MSA and PSP versus IPD, particularly in their initial stages.

## Figures and Tables

**Table 1:** Baseline demographics, clinical features, and a-WBAR.

	Controls *N* = 10	IPD *N* = 20	PSP *N* = 12	MSA *N* = 8	Group comparisons	Significant pairwise comparison
Age (years)^a^ Mean ± SD	64.6 ± 9.9	62.2 ± 11.5	69.9 ± 5.6	60.4 ± 10.9	*F* = 1.96 df = 3 *p* = 0.13	

Gender (M : F)^b^	3 : 7	8 : 12	6 : 6	6 : 2	*F* = 4.1 df = 3 *p* = 0.25	

Disease duration^a^ (years) Mean ± SD	N/A	3.1 ± 3.3	2.2 ± 1.5	3.8 ± 3.6	*F* = 0.75 df = 2 *p* = 0.47	

a-WBAR^a^ (Mean ± SD plus 95% confident interval)	0.37% ± 0.28 (0.17–0.57)	0.54% ± 0.38 (0.32–0.68)	1.26% ± 0.51 (0.95–1.58).	1.65% ± 1.12 (0.71–2.59).	*F* = 12 df = 3 *p* < 0.001	IPD versus MSA < 0.001 IPD versus PSP = 0.004

UPDRS III^c^ (median score plus range)		23.2 ± 12 (3–46)	37 ± 14.5 (20–62)	45.3 ± 13 (29–67)	*χ* ^2^ = 13 df = 2 *p* = 0.001	IPD versus MSA = 0.001 IPD versus PSP = 0.014

CGI-S^c^ (median score plus range)		3.4 ± 0.6 (3–5)	4.4 ± 0.5 (4-5)	4.64 ± 0.5 (4-5)	*χ* ^2^ = 19 df = 2 *p* = 0.001	IPD versus MSA < 0.001 IPD versus PSP < 0.001

H&Y^c^ (median score plus range)		1.9 ± 0.6 (1.0–3.0)	2.6 ± 0.7 (2.0–4.0)	3.0 ± 0.8 (2.0–4.0)	*χ* ^2^ = 12 df = 2 *p* = 0.002	IPD versus MSA = 0.002 IPD versus PSP = 0.011

FAB^c^		14.6 ± 3.5 (5–18)	10.0 ± 5.3 (3–16)	12.0 ± 4.2 (4–16)	*χ* ^2^ = 7.8 df = 2 *p* = 0.016	IPD versus PSP = 0.08

MMSE^c^		26.9 ± 3.7 (14–30)	20.9 ± 10 (3–30)	26.6 ± 2.5 (22–30)	*χ* ^2^ = 3.19 df = 2 *p* = 0.2	

^a^ANOVA test. ^b^Chi square test. ^c^Kruskal-Wallis test and *post hoc* procedure with Mann-Whitney test *p* = 0.05/3 = 0.016. UPDRS III: Unified Parkinson's Disease Rating Scale Part III; H&Y: Hoehn and Yahr Scale; CGI-S: Clinical Global Impression for Disease Severity; FAB: Frontal Assessment Battery; a-WBAR: annual whole-brain atrophy rates. MMSE: Mini-Mental State Examination.

**Table 2:** Baseline, follow-up scores, and estimates of the sample sizes in PSP (*N* = 12) and MSA (*N* = 8).

	Baseline score	Repeat score	Annual change	Number of subjects required per arm (20% reduction)	Number of subjects required per arm (50% reduction)
*PSP*					
Whole-brain atrophy rate, %	N/A^†^	N/A^†^	1.26 ± 0.5	83	14
UPDRS III	37 ± 14.5	39.2 ± 19	2.3 ± 13	168	27
CGI-S	4.4 ± 0.5	4.6 ± 0.8	0.2 ± 0.6	4889	1048
H&Y	2.6 ± 0.7	3.3 ± 0.9^*∗*^	0.7 ± 0.8	631	101
FAB	10 ± 5.3	10.7 ± 7.8	0.7 ± 4.6	22688	3630
*MSA*					
Whole-brain atrophy rate, %	N/A^†^	N/A^†^	1.65 ± 1.1	234	38
UPDRS III	45.3 ± 13	52.6 ± 10	7.1 ± 9.4	921	148
CGI-S	4.6 ± 0.5	5.1 ± 3.5^*∗*^	0.5 ± 0.5	621	100
H&Y	3.1 ± 0.8	3.7 ± 0.4^*∗*^	0.7 ± 0.7	526	85
FAB	12.0 ± 4.2	10.8 ± 4.4	−1.1 ± 3.4	5020	804

^*∗*^Difference between the baseline and repeat score with a *p* value < 0.05 (Wilcoxon's signed rank test). ^†^SIENA gives the whole-brain atrophy rate for image pairs and does not estimate the baseline and follow-up brain volume. All of the values are the mean ± SD.
